# Beyond methane consumption: exploring the potential of methanotrophic bacteria to produce secondary metabolites

**DOI:** 10.1093/ismeco/ycaf030

**Published:** 2025-02-13

**Authors:** Sascha M B Krause, Naomi I van den Berg, Kristof Brenzinger, Hans Zweers, Paul L E Bodelier

**Affiliations:** School of Ecology and Environmental Sciences, East China Normal University, Shanghai, 200241, China; Department of Microbial Ecology, Netherlands Institute of Ecology (NIOO-KNAW), Wageningen, 6700 AB, the Netherlands; Department of Microbial Ecology, Netherlands Institute of Ecology (NIOO-KNAW), Wageningen, 6700 AB, the Netherlands; School of the Biological Sciences, University of Cambridge, Cambridge, CB2 1RX, United Kingdom; Department of Microbial Ecology, Netherlands Institute of Ecology (NIOO-KNAW), Wageningen, 6700 AB, the Netherlands; Department of Microbial Ecology, Netherlands Institute of Ecology (NIOO-KNAW), Wageningen, 6700 AB, the Netherlands; Department of Microbial Ecology, Netherlands Institute of Ecology (NIOO-KNAW), Wageningen, 6700 AB, the Netherlands

**Keywords:** volatiles, methane oxidation, MAG, pure cultures, species interactions

## Abstract

Microbial methane-consuming communities significantly impact biogeochemical processes and greenhouse gas emissions. In this study, we explored secondary metabolites produced by methane-oxidizing bacteria (MOB) and their ecological roles. We analyzed the volatile profiles of four MOB strains under controlled conditions and conducted a meta-analysis using high-quality genomes from 62 cultured MOB strains and 289 metagenome-assembled genomes to investigate their potential for producing secondary metabolites. Results show species-specific volatile production, such as germacrene by *Methylobacter luteus*, which may play a role in the regulation of environmental methane consumption. The meta-analysis revealed that biosynthetic gene clusters (BGCs) for terpenes and β-lactones were more prevalent in the *Methylocystaceae* and/or *Beijerinckiaceae* families, while aryl polyene BGCs were dominant in the *Methylococcaceae* family, reflecting habitat-specific adaptations. These findings advance our understanding of the metabolic capabilities of MOB and underscore the importance of integrating experimental data with genomic and metabolomic analyses to elucidate their ecology, environmental interactions, and contributions to methane cycling.

## Introduction

Microbial communities and their collective metabolic activities in terrestrial and aquatic systems profoundly influence biogeochemical cycling, including greenhouse gas (GHG) fluxes to and from the atmosphere [1]. Methane (CH_4_) is the second most important gas contributing to global warming, having 28 times the global warming potential of carbon dioxide over a 100-year time scale [2, 3]. CH_4_ contributes 17% [2] to total global warming and is generated and emitted mainly by natural and agricultural wetlands, the oil and gas industry, and livestock [2, 4]. Considering its importance but also the large uncertainties in global CH_4_ emissions [5, 6], enhanced understanding of underlying mechanisms of global CH_4_ dynamics is necessary. Hence more profound knowledge of the microbes responsible is necessary, as evidenced by the improvement of earth system models after the incorporation of microbial explicit information [7]. Moreover, recent research has revealed many yet undiscovered properties and biogeochemical traits of known as well as newly isolated microbes involved in CH_4_ cycling [8–12].

A crucial role in balancing global sources and sinks of CH_4_ is laid out for microbial CH_4_ consumption, which can be catalyzed using oxygen as an electron acceptor but is also possible under anaerobic conditions using nitrate, nitrite, and iron as electron acceptors or by reverse methanogenesis in syntropy with sulfate reduction [13–16]. The number of cultivated representatives and associated physiological knowledge is vastly more extensive for aerobic methane-oxidizing bacteria (MOB) which are defined as microbes with the potential to utilize CH_4_ as energy and carbon source [17]. Classically, MOB have been divided into types I and II based on biochemical and physiological properties [18], but this division is currently mainly based on phylogeny. Today MOB can be found in the families *Methylococcaceae* (types Ia & Ib)*, Methylothermaceae* (Ic), *Methylocystaceae* (type IIa), and *Beijerinckiaceae* (type IIb). In addition, MOB has been found in the extremophilic family *Methylacidiphilaceae* [19]. Recently, members of the phylum Actinobacteria have been described to be able to grow on CH_4_ putatively forming type IV MOB [12].

The environmental distribution of aerobic CH_4_-consuming microbes as well as the factors influencing this process have intensively been studied in the past decades [17]. The most recent addition to the list of methanotrophic traits is the ability to reduce minerals under hypoxic conditions [20]. The collective data on environmental distribution, ecology, and physiology has even led to the postulation of conceptual life-history schemes for these organisms [21, 22] classifying the major types of MOB as competitors, stress-tolerators, or ruderals.

The emphasis in environmental studies on MOB was mainly on investigating the effect of environmental factors on the process of CH_4_ oxidation and or on the abundance and diversity of the microbes involved in isolation. Studies on interactions of MOB with other biotic components in the environment are scarce as well as potential mechanisms involved [8]. Important agents in the biotic interaction of microbes are secondary metabolites, which are molecules synthesized to perform functions not related to that organism’s primary metabolism, such as pigments, antibiotics, volatiles, and non-ribosomal peptides producing for example siderophores [23]. Previous investigations into secondary metabolites by MOB have demonstrated that these microbes are capable of producing metabolites allowing for intercellular and interspecies chemical communication [24–26] but also for scavenging copper from the environment [27]. The recently discovered secondary metabolite Tundrenone [26] was shown to be involved in the response to hypoxia of a *Methylobacter* species [28].

Recently, it was demonstrated that volatiles played a central role in the interaction of MOB with heterotrophic bacteria [29]. Volatiles are small organic molecules belonging to the group of secondary metabolites, synthesized, and released by microbes into their ambient environment to fulfill a range of functions, from intercellular communication to antimicrobial warfare, and hence of importance in microbial community ecology and dynamics [30, 31]. However, there are still numerous open questions about when and why microbes are producing these volatiles. This is also the case for MOB, whereas yet only one study reported on sulfurous compounds (Dimethyl disulfide, Dimethyl trisulfide) produced by heterotrophic bacteria potentially playing a role in the observed stimulation as well as inhibition of methanotrophic growth and activity in the presence of a range of heterotrophic bacteria without physical contact to the MOB [29]. In the same study, one of the MOB tested produced terpenes, which potentially serve many functions in interaction and communication with other biota and hence, can have an important influence on CH_4_ consumption in the environment [29].

This study aims at broadening the scope of the role of secondary metabolites for methanotrophic ecology and environmental CH_4_ consumption by assessing volatile production by a representative set of methanotrophic strains under controlled laboratory conditions. In addition, a meta-analysis was included to enhance our understanding of the capacity of MOB to produce secondary metabolites. Therefore, we utilized MOB genomes and metagenome-assembled genome (MAG) information available in public databases to identify secondary metabolite gene clusters and their link to MOB taxonomy and habitat of isolation.

We expect that this analysis will reveal to what extent patterns in secondary metabolites can be explained by shared ancestry or by historical contingency from the habitat of isolation (i.e. convergent evolution of traits), and may shed light on methanotrophic functional diversity. Hence, we aim to predict and elucidate the presence and strength of these patterns across aerobic methanotroph taxonomy and habitat for the genomes of 62 strains of cultured MOB and for the genomes of 289 MAGs from metagenomic studies.

## Material and methods

### Volatile profiles

#### Strain selection and pre-cultivation

The following four strains were selected for this study, on the basis of their variation in putative C1-metabolism pathways and overall variation in types and genera: *Methylocella silvestris* (*Beijerinckiaceae*, type IIb), *Methylosinus trichosporium* (*Methylocystaceae*, type IIa), *Methylomonas sp.* LL1 (*Methylococcaceae*, type Ia), and *Methylobacter luteus* (*Methylococcaceae*, type Ia). Liquid cultures were grown to mid-exponential phase in glass bottles filled with 20 ml nitrate mineral salts (NMS) medium (pH 6.8) on a shaker at 120 rpm at 25°C [29, 32]. NMS medium agar plates were spread with 50 μl of MOB and pre-incubated in gas-tight jars containing 20% CH_4_ headspace at 25°C for 5–8 days due to different timing of growth.

#### Volatile trapping and cell density

##### Liquid cultures

Airtight glass vials of 20 ml with Teflon stoppers were prepared for the experiment. For each treatment, a total of 20 vials were prepared (four replicates for each of the four strains, and four empty vials for the control treatment). Each vial was filled with 16 ml of NMS medium. Cultures growing in the exponential phase were diluted to an optical density (OD_600nm_) of 0.1 (Thermo Scientific™ Genesys 20 spectrophotometer, Breda, The Netherlands). Subsequently, 4 ml of the diluted cultures was added to the vials, locked airtight and enriched with 10.000 ppm_v_ CH_4_, and put on a shaker at 120 rpm in the dark.

After a 24-hour incubation period to capture volatiles within the exponential growth phase, each vial was opened and vented for 30 min in a fume hood to remove all gas from the previous incubation. Afterward, each vial was equipped with a HighSorb probe (Type AxABC, Markes International, Ltd., Llantrisant, UK). This probe consists of a metal probe body fitted with a polydimethylsiloxane (PDMS) absorption phase (HS-P1) which was mounted into the rubber lid of the vial with a needle connected to the non-trapping end of the probe. Vials without inoculated bacteria were serving as controls next to vials with the four different MOB strains. After 4 hours in the dark at 25°C volatile traps were removed, closed, and stored at 4°C until further analysis. Cell density was determined from each individual vial by OD_600nm_ measurements after volatile trapping (Supplementary Table 1) to account for any differences related to cell growth and overall viability.

##### Agar-grown cultures

After pre-incubation, individual plates were opened and vented for 30 min in a fume hood to remove all gas from the previous incubation. Afterward, plates were sealed again and placed in closed flux chambers (V: 172 ml) [33], containing a sampling port with a silicon rubber septum. One percent of CH_4_ (10 000 ppm_v_) was added to the headspace of each chamber and incubated for 48 hours. Afterward, individual plates were opened and vented for 30 min in a fume hood to remove all gas from the previous incubation. Plates were sealed and placed in the same closed flux chambers, and a steel volatile trap containing 150 mg Tenax TA and 150 mg Carbopack B (Markes International, Ltd., Llantrisant, UK) was inserted [34]. Empty agar plates were serving as controls next to agar plates with the four different MOB strains. After 4 hours in the dark at 25°C volatile traps were removed, closed, and stored at 4°C until further analysis. Cell density was determined from each individual plate by OD_600nm_ measurements after volatile trapping (Supplementary Table 1) to account for any differences related to cell growth and overall viability.

#### Volatile profiles and statistical analysis

Trapped volatiles were introduced into the GC-QTOF (model Agilent 7890B GC and the Agilent 7200AB QTOF, Santa Clara, USA). GC-QTOF processing of High sorb as well Tenax traps were performed in accordance with [34, 35].

For the volatolomics analysis, the acquired raw mass spectrometry (MS) data was extracted to m/z format using MassHunter Qualitative Analysis Software V B.07.00 (Agilent Technologies, Santa Clara, CA, USA). The m/z data was processed with MZMine V 2.36 [36] to create a m/z and peak intensity table that could be used as an input file for MetaboAnalyst 4.0 software [37]. Before the statistical analysis, the data was filtered using interquartile range (IQR) and normalized by the log transformation with automatic scaling [35]. To identify significant abundant mass features, one-way-ANOVA with post hoc Tukey test (HSD-test) was performed between the data sets (Supplementary Information Data File 1). To identify important mass features in the samples, Partial Least Squares Discriminant Analysis (PLSDA) was performed. Mass features were statistically relevant if *P*-values were ≤ .05 (Supplementary Information Data File 1). Statistical-relevant mass features were further used for the compound identification. For visualization of the statistically relevant compounds, we used the heatmap function of MetaboAnalyst 4.0.

#### Biosynthetic gene cluster meta-analysis

Strains and MAG were pre-selected from a previous study with a comprehensive list of published MOB genomes and MAGs [38]. We additionally searched both the IMG/M data management and analysis system [39] and the NCBI GenBank database for newly deposited strains and MAGs. Genome information was collected as a nucleotide FASTA file using “datasets” from NCBI Datasets Command-line tools. The correct annotation of MAGs was verified by using the GTDB-Tk software toolkit for assigning taxonomic classifications to microbial genomes based on the Genome Database Taxonomy [40]. Genome quality parameters were assessed by using CheckM [41]. Genomes from MAGs generally were represented by a genome completeness at a 90% level while strain genomes depicted about 98% completeness. The effect on identifying biosynthetic gene clusters (BGC) should be negligible since gaps are likely distributed over the genome rather than being concentrated in a specific area of the genome. In total, 63 MOB genomes and 289 MAG sequences had adequate genome quality (Supplementary Information Data File 1). A list of key information per strain is included in Supplementary Table 2.

Each genome was run through the AntiSMASH 6.1.1 pipeline [42], with standard parameters to identify potential gene clusters of secondary metabolites in each of the studied strains and MAGs. To include a variable for habitat to assess the distribution of habitat types and secondary metabolite clusters, the environment of isolation was retraced from the literature by using “dataformat” from NCBI Datasets Command-line tools (see Supplementary Information Data File 1). These environments were then placed into either of the following categories: “aquatic fresh”, “aquatic marine”, “(upland) soil”, “wetland”, “engineered”, i.e. landfill soils, denitrification tanks, bioreactors, activation sludges, etc., “plant-associated”, “extreme”, i.e. geothermal and hypersaline environments, and “animal (body parts).”

To check whether clusters varied significantly across habitats (environment) and families/MOB types (based on taxonomic affiliations), we used non-metric dimensional scaling (NMDS) and Permutational Multivariate Analysis of Variance (PERMANOVA) using functions from the vegan package (version 2.6.4) in R environment (version 4.2.2) [43]. In some cases, PERMANOVA was unable to produce meaningful results due to high beta-dispersion. These cases were excluded from further analysis.

Since the final table of BGCs is compositional, we pre-treated the data following a procedure developed for high-throughput sequencing data with similar data structures [44]. In brief, we first employed the Bayesian-multiplicative replacement method (cmultRepl) from zCompositions package (version 1.4.1) in R to account for the large number of zero values in the data set. We then used centered log-ratio (CLR) transformation using the Compositions package (version 2.0.5) in R and calculated Aitchison distances. In addition, we also employed a Kruskal–Wallis test and subsequent Dunn post hoc test with Benjamin–Hochberg correction in the R package dunn.test (version 1.3.5) to find which clusters specifically, varied significantly among habitat type and families/MOB type.

## Results

### MOB volatile profiles

**Figure 1:**
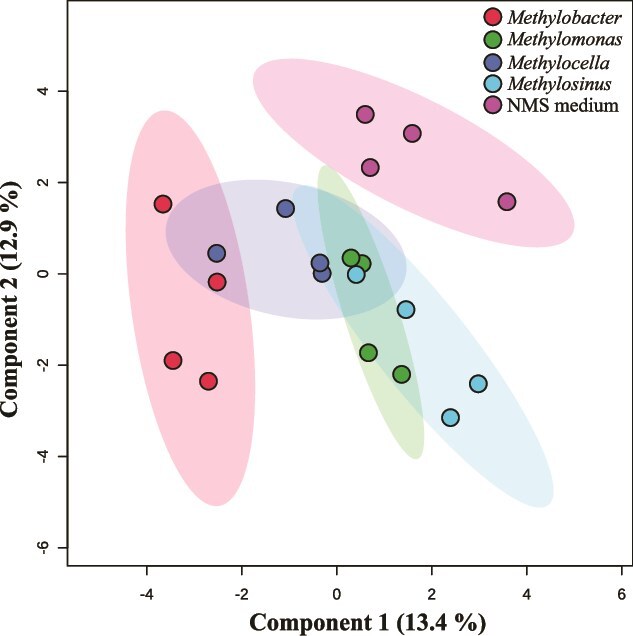
PLSDA 2D-plot of GC–MS data of volatiles emitted from monocultures of MOB strains grown in liquid NMS medium and NMS medium without inoculated bacteria.

The results of the PLSDA demonstrate distinct clustering in the volatile composition of the reference NMS medium without inoculated bacteria and the four tested MOB strains in liquid culture (Fig. 1). The same result was obtained from experimental data with MOB strains grown on NMS medium agar plates (Supplementary Fig. 1).

The volatile profile of *M. luteus* was distinctly different compared to the other stains with only some overlap with *M. silvestris*. The other MOB strains exhibited consistently overlapping volatile profiles across both liquid and solid growth media. In particular, the profiles of *Methylomonas sp.* and *M. trichosporium* were strongly overlapping. Furthermore, the volatile blend of the control was markedly different from that of the biological samples (Fig. 1, Supplementary Fig. 1).

**Figure 2:**
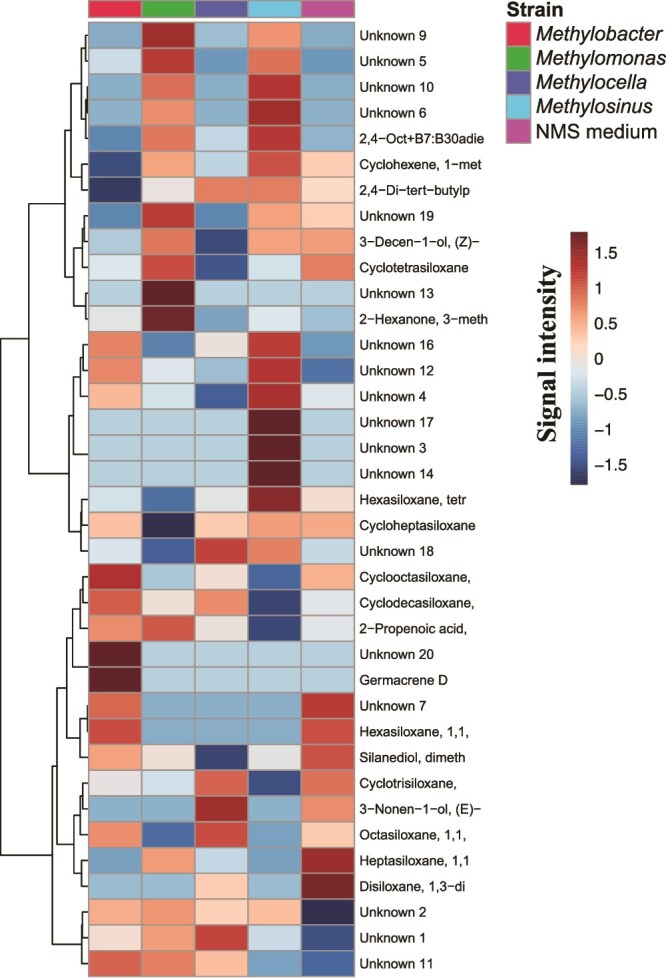
A heatmap displaying average profiles of statistically significant compounds from the volatolomics dataset of MOB strain monocultures grown in liquid NMS medium, compared to NMS medium without bacterial inoculation (*n* = 4). Individual compound profiles for each sample are provided in Supplementary Fig. 2. “Unknown” refers to compounds detected via GC–MS analysis for which no representative matching spectra in databases were available.

A heatmap demonstrated clear patterns in volatile profiles across the four strains grown in an NMS medium (Fig. 2, Supplementary Fig. 2). Regions with consistency across replicates in a volatile intensity, paralleled by low to no detection of the same compound in the reference treatment, are indicative of a strain-specific volatile production. Similarly, regions in which compounds are detected for the reference—indicative of volatiles contained in the medium, without a biosynthetic origin—and not for a strain. We identified germacrene, a compound belonging to the class of terpenes, that was only detected in the volatile profile of *M. luteus* both for cultures grown in liquid (Fig. 2, Supplementary Fig. 2) and on agar plates (Supplementary Fig. 3, Supplementary Fig. 4).

### MOB biosynthetic gene cluster meta-analysis

We first compared the total number of BGCs in MAGs and strain genomes to identify similarities that may result from shared ancestry or historical contingency related to the habitat of isolation/sample origin (Supplementary Tables 3 and 4). Please note, at the family level, we identified an overrepresentation of MAGs belonging to the family *Methylococcaceae* (*n* = 249)*,* while genomes from strains included representatives from all major methanotrophic families and novel representatives within *Methylacidophilaceae* (*n* = 3) and *Mycobacteriaceae* (*n* = 1) (Supplementary Table 3). At the habitat level, we identified an overrepresentation of MAGs from aquatic environments (*n* = 257) but not from many soil environments (*n* = 15). In contrast, strain genomes included a variety of environments (Supplementary Table 4).

**Figure 3:**
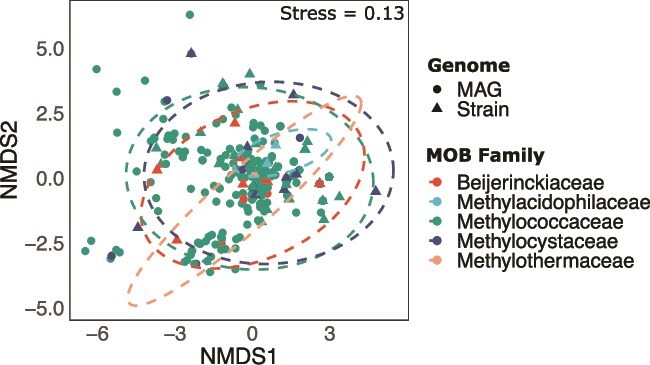
Non-metric multidimensional scaling (NMDS) of BGC compositions from MAG’s and strain genomes at the family level. Ellipses depict 95% confidence intervals for major MOB families. This analysis did not consider BGCs singletons and doubletons across sample sums in a specific BGC cluster.

In Fig. 3, the NMDS plot displays a stress value of 0.13, indicating a reasonably good fit for the dimensional scaling. The MOB families *Beijerinckiaceae*, *Methyloacidophilaceae*, *Methylococcaceae*, and *Methylocystaceae* show overlapping patterns, whereas the family *Methylothermaceae* exhibits a distinctly narrower distribution pattern.

**Figure 4:**
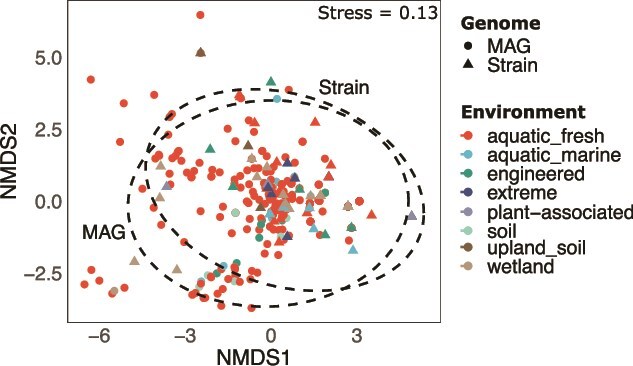
Non-metric multidimensional scaling (NMDS) of BGC compositions from MAG’s and strain genomes at the family level. Black ellipses depict 95% confidence intervals for MAG and strain genomes, respectively. This analysis did not consider BGCs singletons and doubletons across sample sums in a specific BGC cluster.

Focusing on the impact of historical contingency, NMDS shows no clear separation between the two groups, despite PERMANOVA revealing a statistically significant difference in BGC compositions between MAGs and strain genomes (*F* = 3.26, *P* = .005). This result is likely driven by the unequal sample sizes (63 strain genomes vs. 289 MAGs) and the overrepresentation of aquatic samples indicating that the overall patterns remain largely similar (Fig. 4).

In our final analysis, we aimed to identify which BGCs can be linked to specific “Habitat” (historical contingency) or “Family” (shared ancestry) predictors. Overall the statistical analysis results show that the variable “Family” explained more cluster variation in both MAGs and strain genomes (Tables 1 and 2).

**Table 1:** Secondary metabolite gene clusters that differed significantly (Kruskal–Wallis test, *P* < .05) in occurrence and/or number of copies across the variable habitat and family (all categorical) of the studied MAGs, and which categories within each variable contrasted significantly (Dunn test with Benjamin Hochberg correction for multiple comparisons, *P* < .05). Only main families were analyzed (*N* > 2). Underlined are observations (partially) consistent between MAGs and strain genomes. Numbers in “Gene clusters” correspond to numbers in “Interpretation”.

**Variable**	**Gene clusters**	**Interpretation**
**Habitat**	1. Terpene 2. Aryl polyene 3. Type I polyketide 4. Type III polyketide 5. RIPP^a^ like (Bacteriocin) 6. Phenolic lipids 7. Hserlactone 8. NAPAA^b^ 9. β-lactone 10. LAP^c^	1. Occurrence higher in wetlands and soils than in aquatic ecosystems 2. Occurrence higher in soils than in freshwater aquatic ecosystems 3. Occurrence higher in freshwater than marine aquatic ecosystems 4. Occurrence lower in aquatic ecosystems than in all other environments 5. Occurrence higher in wetlands than in all other environments and higher in engineered than aquatic ecosystems 6. Occurrence higher in soils than in any other ecosystems 7. Occurrence higher in soils than in freshwater aquatic ecosystems 8. Occurrence higher in wetlands than in aquatic ecosystems 9. Occurrence higher in wetlands and marine aquatic ecosystems than in freshwater aquatic, engineered, and soil ecosystems 10. Occurrence higher in wetlands than in all other environments
**Family**	1. Terpene 2. Aryl polyene 3. Type I polyketide 4. Type III polyketide 5. RIPP^a^ like (Bacteriocin) 6. Hserlactone 7. Carotenoid 8. NAPAA^b^ 9. β-lactone 10. LAP^c^	1. Occurrence higher in *Methylocystaceae and Beijerinckiaceae* than in *Methylococcaceae* 2. Occurrence higher in *Methylococcaceae* than any other 3. Occurrence higher in *Methylococcaceae* than *Methylocystaceae* 4. Occurrence higher in *Methylocystaceae* than in *Methylococcaceae* 5. Occurrence higher in *Methylococcaceae and Methylocystaceae* than *Beijerinckiaceae* 6. Occurrence higher in *Beijerinckicaceae* and *Methylocystaceae* than in *Methylococcaceae* 7. Occurrence higher in *Methylocystaceae* than in any other, higher in *Beijerinckiaceae* than in *Methylococcaceae* 8. Occurrence higher in *Beijerinckicaceae* and *Methylocystaceae* than *Methylococcaceae* 9. Occurrence higher in *Methylocystaceae* than in *Methylococcaceae* 10. Occurrence higher in *Methylocystaceae* than in *Beijerinckicaceae* and *Methylococcaceae*

a ribosomally synthesized and post-translationally modified peptide product

b non-alpha poly-amino acids like e-Polylysin

c Linear azol(in)e-containing peptides

**Table 2:** Secondary metabolite gene clusters that differed significantly (Kruskal–Wallis test, *P* < .05) in occurrence and/or number of copies across the variable habitat and family (all categorical) of the studied strains, and which categories within each variable contrasted significantly (Dunn test with Benjamin Hochberg correction for multiple comparisons, *P* < .05). Only main families were analyzed (*N* > 2). Underlined are observations (partially) consistent between MAGs and strain genomes. Numbers in “Gene clusters” correspond to numbers in “Interpretation”.

**Variable**	**Gene clusters**	**Interpretation**
**Habitat**	1. Type III polyketide 2. RIPP^a^ like (Bacteriocin) 3. Ectoine	1. Occurrence higher in extreme ecosystems than in other ecosystems 2. Occurrence higher in freshwater aquatic than in extreme ecosystems 3. Occurrence higher in strains isolated from marine aquatic than soil, plant-associated, engineered, and wetland ecosystems; lower than aquatic freshwater ecosystems
**Family**	1. Terpene 2. Aryl polyene 3. Type I polyketide 4. Type III polyketide 5. RIPP^a^ like (Bacteriocin) 6. NRPS^b^ like 7. NAPAA^c^ 8. β-lactone	1. Occurrence higher in *Methylocystaceae and Methylacidophilaceae* than in *Methylococcaceae* 2. Occurrence higher in *Methylococcaceae* than any other 3. Occurrence higher in *Beijerinckicaceae* than in any other 4. Occurrence higher in *Methyloacidophilaceae* than in *Methylococcaceae* 5. Occurrence higher in *Methylocystaceae* than *Methyloacidophilaceae* and *Methylococcaceae* 6. Occurrence higher in *Beijerinckicaceae* and *Methylocystaceae* than *Methylococcaceae* 7. Occurrence higher in *Methyloacidophilaceae* than in any other 8. Occurrence higher in *Methylocystaceae* than in *Methylococcaceae*

a ribosomally synthesized and post-translationally modified peptide product

b non-ribosomal peptide synthase

c non-alpha poly-amino acids like e-Polylysin

Some cluster variations between MAGs and strain genomes were explained by the same BGCs within the “Family” predictor, such as terpene, aryl polyene (bacteriocin), and β-lactone. Additionally, the predictor “Habitat” explained more cluster variation for MAGs (10 clusters) compared to strain genomes (three clusters). Table 3 presents an overview of identified secondary metabolite gene clusters and their functions, which differed significantly in occurrence and/or number of copies across the “Habitat” and “Family” variables of the studied strains and MAGs.

**Table 3:** Overview of identified secondary metabolite gene clusters and their functions that differed significantly (Kruskal–Wallis test, *P* < .05) in occurrence and/or number of copies across the variable habitat and family of the studied strains and MAGs.

**Name**	**Cluster full name**	**Description of function**
**Terpene**	Terpene	Odoriferous metabolites are generally associated with plant or fungal origins. Terpenes are involved in intercellular communication [45]
**Aryl polyene**	Aryl polyene	Bacterial pigment involved in protecting against photobiological damage [46]; is functionally related to antioxidative carotenoids [47].
**Type I polyketide**	Type I polyketide synthases	A class of polyketides that are typified by very long carbon chains that are mostly linear; frequently co-occurring with non-ribosomal peptide synthetases [48]; Many polyketides have antimicrobial and immunosuppressive properties [49].
**Type III polyketide**	Type III polyketide synthases	Synthesis mechanism is non-linear (iterative) in which the active sites are reused repeatedly. Many polyketides have antimicrobial and immunosuppressive properties [49].
**RIPP like (Bacteriocin)**	Ribosomally synthesized and post-translationally modified peptide product	Ribosomal synthesized antimicrobial peptides [50].
**Phenolic lipids**	Phenolic lipids	These compounds can inhibit bacterial, fungal, protozoan, and parasite growth but depend on protein interactions and/or their disruptive effect on cellular membranes [51].
**Hserlactone**	Homoserine lactone	A class of signaling molecules involved in bacterial quorum sensing, enabling cells to regulate gene expression depending on population density, and hence comprising an important element to intercellular communication (Fuqua *et al.*, 2001).
**NAPAA**	Non-alpha poly-amino acids like epsilon -Poly-L-lysin	Has been demonstrated to show antimicrobial activity against both Gram-positive and -negative bacteria [52]
**β-lactone**	β-lactone	Compounds containing β-lactone are chemically diverse, many of which are characterized by potent bioactivity against bacteria and fungi [53].
**LAP**	Linear azol(in)e-containing peptides	A subfamily of RiPPs [54]. This family of RiPPs exhibits various bioactivities, such as DNA gyrase inhibitors and hemolytic factors [55], and can include structurally rigid compounds with selective antimicrobial activity [56].
**Carotenoid**	Carotenoid	Carotenoids play a pivotal role in bacterial physiology by providing protection against UV radiation and mitigating oxidative stress [57].
**Ectoine**	Ectoine	Ectoine is synthesized primarily by halophilic bacteria and confers resistance toward osmotic stress induced by high ambient salt and temperature. Ectoine has been shown to stabilize enzymes [58].
**NRPS like**	Non-ribosomal peptide synthase	Synthesizes peptides independently of mRNA. Clusters can include families of cyclic lipopeptide antibiotics, families of cyclic hexapeptide protease inhibitors, phenolic lipids, and a wide range of other peptides that are not synthesized by ribosomes. Most non-ribosomal peptides are often toxins, siderophores, or pigments [59].

## Discussion

This study highlights the potential of CH_4_-oxidizing bacteria (MOB) to produce diverse secondary metabolites. While MOB can produce volatiles in both the liquid and gaseous phases, this study focuses on gaseous-phase volatiles because they potentially have a larger range of impact on species interactions within the soil pore space. We assessed volatile production in the pure cultures of *M. silvestris* (*Beijerinckiaceae*, type IIb), *M. trichosporium* (*Methylocystaceae*, type IIa), *Methylomonas sp.* LL1 (*Methylococcaceae*, type Ia), and *M. luteus* (*Methylococcaceae*, type Ia) under controlled conditions. We further conducted a meta-analysis using genomic data from cultured strains and metagenomic studies to explore the genetic potential of MOB for secondary metabolite production and their broader functional and ecological implications.

The volatolomics of four MOB strains representative of three major phylogenetic lineages consistently show distinct volatile profiles for cultures grown in liquid NMS medium (Fig. 1, Supplementary Fig. 2) and on plates (Supplementary Figs 3 and 4). Despite the distinct clustering in volatile profiles across the four studied strains, the amount of variation explained by the two dominant axes was limited, suggesting overall limited biogenic modification of the volatosphere in our incubations. Specifically, only 37 (in liquid cultures) or 28 (in plate-grown cultures) out of >2000 distinct volatile compounds were significantly different between live cultures and the non-inoculated media or agar plates.

This study demonstrated that aerobic MOB produce distinct volatile blends, even within the same taxonomic family, as illustrated by the differing volatile profiles of *M. luteus* and *Methylomonas sp*. LL1 (Fig. 1). In *M. luteus*, germacrene, a sesquiterpene, was consistently produced in both liquid and plate-grown cultures but was absent in the other MOB strains. Notably, a BGC associated with terpene synthesis was identified in this organism [29], providing a potential genomic basis for its volatile production. However, most detected volatiles could not be directly linked to specific BGCs, highlighting the inherent complexity of secondary metabolite biosynthesis and the limitations of genomic predictions without complementary transcriptomic or proteomic validation.

Terpenes may also play a role in CH_4_ oxidation, either as inhibitors or signaling compounds. Monoterpenes, for instance, have been shown to inhibit CH_4_ oxidation in methanotrophic cultures [60] and in forest soils, where terpenes are derived from pine tree roots or decomposing litter [61]. By producing terpenes, specific MOB species may inhibit and thereby outcompete other MOB species or ammonia oxidizers for CH_4_ and O_2_, or even use terpenes as antibiotics. However, inhibitory effects of sesquiterpenes on CH_4_ oxidation have yet to be demonstrated and highlight the need to examine the ecological functions of terpenes in CH_4_ cycling and their broader implications for GHG emissions.

Terpenes, like Germacrene, have been shown to play an important role in direct trophic interactions between soil bacteria and protists. For instance, Schulz-Bohm *et al.* [62] demonstrated that bacterial mutants unable to express a terpene synthase lost the ability to affect protists. In a previous study, Murase and Frenzel [63] estimated the number of methanotroph-feeding protists in a rice field soil by determining the most probable number (MPN) using MOB as food bacteria. It was demonstrated that several members of the family *Methylocystaceae* were significantly less edible by protists than *M. luteus*. Protists feeding on this MOB yielded MPN counts like the common food bacteria *Escherichia coli*. The authors did not have an explanation at the time for the selective grazing, but our findings can add an interesting new perspective. The production of germacrene by *M. luteus* may be volatile organic carbons (VOCs)-mediated precursor providing information about suitable prey to soil protists, highlighting a promising opportunity for further research.

It is tempting to associate volatiles produced by MOB with decomposition processes in soils and peat, as these environments release a variety of volatile organic compounds, including substrates relevant to MOB such as CH_4_ and methanol [64, 65]. However, it remains challenging to understand how volatile production by MOB exerts ecological effects amidst volatile emissions from other sources. Elucidating the ecological role of volatile production by MOB will require carefully planned mechanistic experiments, the necessity of which is supported by the findings of our study.

The meta-analysis highlights a strong phylogenetic signal in BGC distribution, with shared ancestry playing a dominant role in shaping secondary metabolite potential, underscoring the differences found in VOC profiles with pure cultures of MOB. This is not surprising because core biosynthetic pathways are more likely to be inherited vertically and conserved across generations, even though environmental factors and horizontal gene transfer can still influence the diversification and adaptation of these pathways to specific ecological niches. [66].

For example, the meta-analysis showed that *Methylocystaceae* genomes encoded consistently more β-lactone BGCs than *Methylococcaceae* genomes. These BGCs can have antimicrobial properties and lactone motifs are key structural components of small signaling molecules, indicating their involvement in signaling processes within microbial communities [67]. In contrast, *Methylococcaceae* genomes frequently harbor aryl polyene BGCs, associated with protection against visible light in oxygenated environments, such as surface water and sediments [46]. Another example of habitat contingency driving the difference between MOBs belonging to the family *Methylococcaceae* and *Beijerinckiaceae* is the significantly higher occurrence of gene clusters for type I polyketides in the *Beijerinckiaceae* family of pure culture genomes. This gene cluster, when expressed, confers antimicrobial properties to the MOB, and may be beneficial in environments competitive for CH_4_, O_2_, and minerals, such as forest/upland soils and wetlands soils that these strains tend to occur in [3, 49]. However, the analysis of MAG did not support this observation but could be a result of a sampling bias. There are 249 MAG of the *Methylococacceae* family versus 10 MAG of the *Beijerinckiaceae* family in the MAG data set (Supplementary Tab. 3).

Overall, these results suggest that the distribution of BGC represents a set of genomic traits characteristic of distinct life strategies that fit into the Competitor-Stress Tolerator-Ruderal functional classification framework applied to MOB earlier [21, 68]. In this framework, BGCs in the family *Methylococcaceae* can be associated with the competitor-ruderal category (opportunists), and with the stress tolerator category for the families *Methylocystaceae* and *Beijerinckiaceae*.

While our study provides evidence for distinct volatile profiles among MOB strains, the integration of volatile profiling with BGC predictions remains a challenging task. For example, BGCs for terpenes were significantly less prevalent in the *Methylococcaceae* family compared to the *Methylocystaceae* and *Beijerinckiaceae* families. However, the actual production of terpenes, such as germacrene, was only observed in *M. luteus*, highlighting a discrepancy between genomic potential and realized metabolite production. The “unknown” volatile compounds detected in our experiments may also include terpenoid compounds, but further validation is required to confirm their identities.

This study highlights distinct volatile profiles and biosynthetic potential among aerobic MOB, but certain limitations must be acknowledged. To truly integrate genomics and metabolomics (of a single species), it is essential to continue exploring gene function through methods such as gene knockout studies on non-model organisms, which can increase annotation specificity. Additionally, enhancing methods for compound identification and functional validation remains a critical need. External databases like mVOC 4.0 [69] and the BGC Atlas [70] could aid in volatile identification, ecological context, and linking BGCs to volatile production. To address the discrepancy between secondary metabolite production potential and realized production, future studies could benefit from transcriptomics or proteomics data measured for different MOB types.

Sampling bias and limited metadata for certain strains pose additional challenges. For example, the overrepresentation of aquatic freshwater environments in the MAG dataset may skew results, as seen in the underrepresentation of the *Beijerinckiaceae* family. Similarly, reliance on isolation data from decades-old strains may not accurately reflect habitat preferences for entire genera or species. We accounted for this problem by including MAGs to have annotated genomes of newly sequenced strains that could shed a more conclusive light on secondary metabolites in aerobic MOB. Finally, our results of the meta-analysis may be limited by the annotation accuracy of the pipeline used.

## Conclusion

In this study, we demonstrate distinct volatile profiles among a representative set of aerobic MOB strains, indicating species-specific production of secondary metabolites. Particularly notable was the production of germacrene by *M. luteus*, which together with earlier findings of production of other terpenes by this species suggest a potential role of these compounds in CH_4_ biogeochemistry and or biotic interactions with implication for GHG emissions from various ecosystems. The comprehensive meta-analysis highlighted a differential distribution of BGCs across methanotrophic families, reflecting their adaptation to distinct environmental niches, and highlighted genomic traits linked to secondary metabolite production are aligned with the Competitor-Stress Tolerator-Ruderal functional classification framework for MOB.

While this study advances our understanding of MOB secondary metabolites, it also points to the need for enhanced gene function annotation, comprehensive omics approaches but also advanced chemical analytics, to bridge the gap between genomic potential and actual metabolite production. However, our findings provide a comprehensive foundation regarding the biosynthetic potential of MOB to generate compounds with implications for CH_4_ cycling. The data also presented the basis for further studies into CH_4_ cycling microbes and modes of interaction with their environment, which are vital to better understanding uncertainties in global CH_4_ emissions.

## Supplementary Material

003a_SupplementaryInformation_accepted_ycaf030(1)

003b_SupplementaryInformation_DataFile1_accepted

## Data Availability

All data generated or analyzed during this study are available in this published article and its supplementary information files, except for the primary raw MS data. This dataset is securely stored in the internal data portal of the Netherlands Institute of Ecology, which is not publicly accessible. However, access can be granted upon request by contacting the corresponding author.
