# Supplementary material for: Beyond methane consumption: exploring the potential of methanotrophic bacteria to produce secondary metabolites
Source: ISME Commun. 2025 Feb 13;5(1):ycaf030. doi: 10.1093/ismeco/ycaf030 (PMC11964084; doi:10.1093/ismeco/ycaf030)
Supplement: 003a_SupplementaryInformation_accepted_ycaf030(1) [file 003a_supplementaryinformation_accepted_ycaf030(1).pdf]

Supplementary Materials for:

Beyond methane consumption: Exploring the potential of methanotrophic bacteria to produce secondary metabolites

Sascha M.B. Krause [1,2], Naomi Iris van den Berg[2,3], Kristof Brenzinger[2], Hans Zweers[2], Paul L.E. Bodelier[2]

[1]School of Ecology and Environmental Sciences, East China Normal University, Shanghai, China

[2]Department of Microbial Ecology, Netherlands Institute of Ecology (NIOO-KNAW), Wageningen, the Netherlands

[3]School of the Biological Sciences, University of Cambridge, Cambridge, United Kingdom

This supplementary file includes:

Supplementary Table 1: Cell densities at the time of volatile trapping

Supplementary Table 2: Information on all 62 strains included in this cross-genomic secondary metabolite analysis. All contigs contain protein-encoding genes (PEGs)

Supplementary Table 3: Average biosynthetic cluster counts in metagenome assembled genomes (MAGs) and pure culture genomes (Strains) grouped at the MOB family level. Alongside total genome counts for each family group are shown. Different levels of genome completeness were incorporated to enhance study and genome diversity as well as to increase the total number of included genomes. Standard deviations are shown, if applicable.

Supplementary Table 4: Average biosynthetic cluster counts in metagenome assembled genomes (MAGs) and pure culture genomes (Strains) grouped by environment. Alongside total genome counts for each environment group are shown. Different levels of genome completeness were incorporated to enhance study and genome diversity as well as to increase the total number of included genomes. Standard deviations are shown, if applicable.

Supplementary Figure 1: PLSDA 2D-plot of GC-MS data of volatiles emitted from monocultures of MOB strains grown on NMS medium agar plates and NMS medium without inoculated bacteria.

Supplementary Figure 2. A heatmap of statistically significant compounds measured in the volatolomics dataset, displaying individual sample profiles of MOB strains grown in liquid NMS medium compared to NMS medium without bacterial inoculation. The heatmap includes hierarchical clustering to group similar compounds and samples. 'Unknown' refers to compounds detected via GC-MS analysis that did not match any representative spectra in the reference databases used.

Supplementary Figure 3. A heatmap displaying average profiles of statistically significant compounds measured in the volatolomics dataset of monocultures of MOB strains grown on NMS medium agar plates, compared to NMS medium agar plates without inoculated bacteria (n=4). 'Unknown' refers to compounds identified in the GC-MS analysis but without a representative standard at the specific retention time values. 'Unknown' refers to compounds detected via GC-MS analysis that did not match any representative spectra in the reference databases used.

Supplementary Figure 4. A heatmap of statistically significant compounds measured in the volatolomics dataset, displaying individual sample profiles of MOB strains on NMS medium agar plates, compared to NMS medium agar plates without inoculated bacteria. The heatmap includes hierarchical clustering to group similar compounds and samples. 'Unknown' refers to compounds detected via GC-MS analysis that did not match any representative spectra in the reference databases used.

Supplementary Table 1: Cell densities at the time of volatile trapping

**Strain**	**Medium type**	**OD_{600}**
Methylomonas sp. LL1	Agar	0.53 ± 0.02
	Liquid	0.47 ± 0.05
Methylosinus trichosporium	Agar	0.09 ± 0.05
	Liquid	0.61 ± 0.02
Methylobacter luteus	Agar	0.08 ± 0.03
	Liquid	0.35 ± 0.03
Methylocella silvestris	Agar	0.25 ± 0.03
	Liquid	0.25 ± 0.04

Supplementary Table 2: Information on all 62 strains included in this cross-genomic secondary metabolite analysis. All contigs contain protein-encoding genes (PEGs).

Family	Species/strain	Contigs	Sequence accession	Habitat of isolation	Genome size Mb	References
Methylococcaceae	*Methylococcus capsulatus str. Bath*	1	GCA_000008325.1	Soil, water, sewage, mud, and lake sediments	3.30	Ref. 1, ref. 2
Methylococcaceae	*Methylomonas methanica str. MC09*	1	GCA_000214665.1	Coastal seawater	5.05	Ref. 3
Methylococcaceae	*Methylomonas koyamae str. JCM16701*	382	GCA_001312005.1	Floodwater rice paddy field	4.95	Ref. 4
Methylococcaceae	*Methylomonas denitrificans str. FJG1*	1	GCA_000785705.2	Aquatic freshwater	5.17	Ref. 5. ref. 6
Methylococcaceae	*Methylomonas sp. str. LL1*	2	GCA_015711015.1	River floodplain soil	4.92	Ref. 7
Methylococcaceae	*Methylomonas lenta str. R-45370*	171	GCA_001644015.1	Denitrification tank of a wastewater treatment plant	4.70	Ref. 6, ref. 8
Methylococcaceae	*Methylomonas sp. str. DH-1*	1	GCA_001644685.1	Activated sludge from brewery	4.85	Ref. 9, ref. 10
Methylococcaceae	*Methylomonas sp. str. 11b*	1	GCA_000515215.1	Lake Washington sediment	5.41	Ref. 11
Methylococcaceae	*Methylomonas sp. str. MK1*	5	GCA_000365425.1	Lake Washington sediment	5.23	Ref. 11
Methylococcaceae	*Methylobacter luteus str. IMV-B-3098*	4	GCA_000427625.1	Mouth of a cow	5.03	Ref. 12
Methylococcaceae	*Methylobacter marinus str. A45*	2	GCA_000383855.1	Seawater near sewage outfall	4.99	Ref. 13
Methylococcaceae	*Methylobacter tundripaludum str. SV96*	3	AEGW00000000.2	High arctic wetland soil	4.85	Ref. 14
Methylococcaceae	*Methylobacter sp. str. 21/22*	1	GCA_000685925.1	Lake Washington sediment	4.67	Ref. 11
Methylococcaceae	*Methylobacter sp. str. 31/32*	2	GCA_000733835.1	Lake Washington sediment	5.05	Ref. 11
Methylococcaceae	*Methylobacter sp. str. KS41*	4	GCA_002862125.1	Acidic forest soil	4.71	Ref. 15
Methylococcaceae	*Methylobacter sp. str. BBA5.1*	91	GCA_000746145.1	Surface layer of estuary sediment	5.07	Ref. 13
Methylococcaceae	*Methylomicrobium alcaliphilum str. 20Z*	2	GCF_000968535.2	Sediment from saline soda lakes	4.80	Ref. 16, ref. 17
Methylococcaceae	*Methylomicrobium agile str. ATCC35068*	4	GCF_000733855.1	Wetland sediment samples	4.53	Ref. 12
Methylococcaceae	*Methylomicrobium buryatense str. 5G*	2	GCA_000341735.1	Bottom sediment soda lakes	5.07	Ref. 18

Methylococcaceae	*Methylomicrobium sp. str. Wino1*	1	GCA_003932755.1	Marine sediment	5.06	Ref. 19
Methylococcaceae	*Methylomicrobium album str. BG8*	3	GCA_000214275.3	Muddy water	4.45	Ref. 20
Methylococcaceae	*Methylosarcina fibrata str. AML-C10*	2	GCA_000372865.1	Upper soil layer of a landfill	4.98	Ref. 12
Methylococcaceae	*Methylosarcina lacus str. LW14*	1	GCA_000527095.1	Lake Washington sediment	4.38	Ref. 11
Methylococcaceae	*Methylocaldum szegediense str. O-12*	2	GCA_000427385.1	Manure	5.02	Ref. 21
Methylococcaceae	*Methylocaldum marinum str. S8*	1	GCA_003584645.1	Marine sediment	6.09	Ref. 22
Methylococcaceae	*Methylocaldum sp. str. 14B*	85	GCA_002005105.1	Anaerobic digester (SS-AD reactors)	5.62	Ref. 23
Methylococcaceae	*Methyloglobulus morosus str. KoM1*	183	GCA_000496735.2	Profundal sediment sludge	4.14	Ref. 24
Methylococcaceae	*Methyloprofundus sedimenti str. WF1*	6	GCA_002072955.1	Marine sediment	4.29	Ref. 25
Methylococcaceae	*Methylomarinum vadi str. IT-4*	1	GCA_000733935.1	Hydrothermal sediment	4.34	Ref. 26
Methylococcaceae	*Methylovulum psychrotolerans str. HV10_M2*	1	GCA_002209385.1	Plant material from high-altitude peat bog	4.92	Ref. 27
Methylococcaceae	*Methylovulum miyakonense str. HT12*	1	GCA_000384075.1	Forest soil	4.69	Ref 12, ref. 28
Methylococcaceae	*Methylomagnum ishizawai str. 175*	8	GCA_900155475.1	Rice rhizosphere	5.48	Ref. 29, ref. 30
Methylococcaceae	*Methyloterricola oryzae str. 73AT*	73	GCA_000934725.1	Rice rhizosphere	4.90	Ref. 29
Methylothermaceae	*Methylohalobius crimeensis str. 10Ki*	5	GCA_000421465.1	Sediment from hypersaline lakes	3.51	Ref. 31
Methylocystaceae	*Methylosinus trichosporium str. OB3b*	3	GCA_000178815.2	Soil, fresh water sediments, groundwater	4.96	Ref. 32
Methylocystaceae	*Methylosinus sp. str. LW3*	5	GCA_000527115.1	Lake Washington sediment	5.09	Ref. 11
Methylocystaceae	*Methylosinus sp. str. LW4*	3	GCA_000379125.1	Lake Washington sediment	4.82	Ref. 11
Methylocystaceae	*Methylosinus sp. str. PW1*	12	GCA_000745215.1	Lake Washington sediment	5.13	Ref. 11
Methylocystaceae	*Methylocystis bryophila str. S285*	2	GCA_002117405.1	Wetland peat soil	4.71	Ref. 33, ref. 34
Methylocystaceae	*Methylocystis parvus str. OBBP*	108	GCA_000283235.1	Denitrifying bioreactor (origin closest related strain)	4.48	Ref. 35, ref. 36
Methylocystaceae	*Methylocystis rosea str. SV97*	2	GCA_000372845.1	Arctic wetland soil	3.91	Ref. 37

Methylocystaceae	*Methylocystis sp. str. SC2*	1	GCA_000304315.1	Highly polluted acquifer	3.77	Ref. 38
Methylocystaceae	*Methylocystis sp. str. Rockwell*	7	GCA_000188155.3	Acquifer	4.73	Ref. 39
Methylocystaceae	*Methylocystis sp. str. LW5*	6	GCA_000685825.1	Lake Washington sediment	4.76	Ref. 11
Methylocystaceae	*Methylocystis heyeri str. H2T*	12	GCA_004802635.1	Acidic peat bog soil	4.69	Ref. 40., ref. 41
Methylocystaceae	*Methylocystis hirsuta str. CSC1*	4	GCA_003722355.1	Acquifer	4.21	Ref. 42
Methylocystaceae	*Methylotetracoccus oryzae str. C50C1*	42	GCA_006175985.1	Rice paddy field soil	4.84	Ref. 43
Methylocystaceae	*Methylosinus sporium str. DSM 17706*	55	GCA_003113265.1	Rice paddy	3.79	Ref. 44
Methylocystaceae	*Methylosinus sporium str. SM89A*	161	GCA_007004125.1	Rice rhizosphere	4.59	Tani, unpublished report
Methylocystaceae	*Methylocystis silviterrae*	65	GCF_013350005.1	Boreal forest soil	3.85	Ref. 45
Beijerinckiaceae	*Methylocella silvestris str. BL2*	1	GCA_000021745.1	Acidic forest soil	4.31	Ref. 46, ref. 47
Beijerinckiaceae	*Methylocella silvestris str. TVC*	80	GCA_002891535.1	Permafrost sediment	4.29	Ref. 48
Beijerinckiaceae	*Methylocella tundrae*	1	GCA_900749825.1	Wetland peat soil	3.91	Ref. 49
Beijerinckiaceae	*Methylocapsa acidiphila str. B2*	2	GCA_000427445.1	Wetland peat soil	4.10	Ref. 50, ref. 51
Beijerinckiaceae	*Methylocapsa aurea str. KYGT*	34	GCA_000746085.1	Upland forest soil	3.97	Ref. 52
Beijerinckiaceae	*Methylocapsa palsarum str. NE2*	78	GCA_900114285.1	Subarctic wetland soil	4.11	Ref. 53
Beijerinckiaceae	*Methylocapsa gorgona str. MG08*	1	GCA_004564215.1	Landfill soil	3.3	Ref. 54
Beijerinckiaceae	*Methyloferula stellata str. AR4T*	1	GCA_000385335.1	Wetland peat soil	4.24	Ref. 55
Methylacidophilaceae	*Methylacidiphilum infernorum str. V4*	1	GCA_000019665.1	Hydro/geothermal vent	2.29	Ref. 55
Methylacidophilaceae	*Methylacidiphilum fumariolicum str. SolV*	1	GCA_000953475.1	Hydro/geothermal vent	2.48	Ref. 57
Methylacidophilaceae	*Methylacidiphilum kamchatkense str. Kam1*	41	GCA_000817245.1	Hydro/geothermal vent	2.21	Ref. 58
Mycobacteriaceae	*Candidatus* Mycobacterium methanotrophicum	2	GCF_023515995.1	Sulfur cave	5.00	Ref. 59

Supplementary Table 3: Average biosynthetic cluster counts in metagenome assembled genomes (MAGs) and pure culture genomes (Strains) grouped at the MOB family level. Alongside total genome counts for each family group are shown. Different levels of genome completeness were incorporated to enhance study and genome diversity as well as to increase the total number of included genomes. Standard deviations are shown, if applicable.

	**>90% completeness (MAGs)**		**>98% completeness (MAGs)**		**>98% completeness (Strains)**	
**Family**	**Clusters**	**Studies**	**Clusters**	**Studies**	**Clusters**	**Studies**
Methylococcaceae	8 ± 7	249	7 ± 4	75	11 ±4	33
Methylothermaceae	8 ± 0	2	8	1	10	1
Methylocystaceae	8 ± 4	26	12 ± 5	8	14 ± 4	16
Beijerinckiaceae	7 ± 4	10	10 ± 4	4	13 ± 3	8
Methylacidophilaceae	0	0	0	0	8 ± 1	3
Mycobacteriaceae	0	0	0	0	13	1
Crenotrichaceae	6 ± 1	2	7	1	0	0

Supplementary Table 4: Average biosynthetic cluster counts in metagenome assembled genomes (MAGs) and pure culture genomes (Strains) grouped by environment. Alongside total genome counts for each environment group are shown. Different levels of genome completeness were incorporated to enhance study and genome diversity as well as to increase the total number of included genomes. Standard deviations are shown, if applicable.

	**>90% completeness (MAGs)**		**>98% completeness (MAGs)**		**>98% completeness (Strains)**	
**Family**	**Clusters**	**Studies**	**Clusters**	**Studies**	**Clusters**	**Studies**
Aquatic (freshwater)	8 ± 7	236	7 ± 4	69	13 ± 4	18
Aquatic (marine)	5 ± 3	21	5 ± 2	6	14 ± 5	6
Engineered	10 ± 5	15	10 ± 4	8	10 ± 3	7
Extreme	8 ± 1	2	7	1	9 ± 2	8
Soil	9 ± 4	12	11 ± 4	5	0	0
Upland soil	0	0	0	0	13 ± 4	5
Wetland	12 ± 2	3	0	0	11 ± 3	13
Plant-associated	0	0	0	0	14 ± 4	4
Animal (body parts)	0	0	0	0	15	1

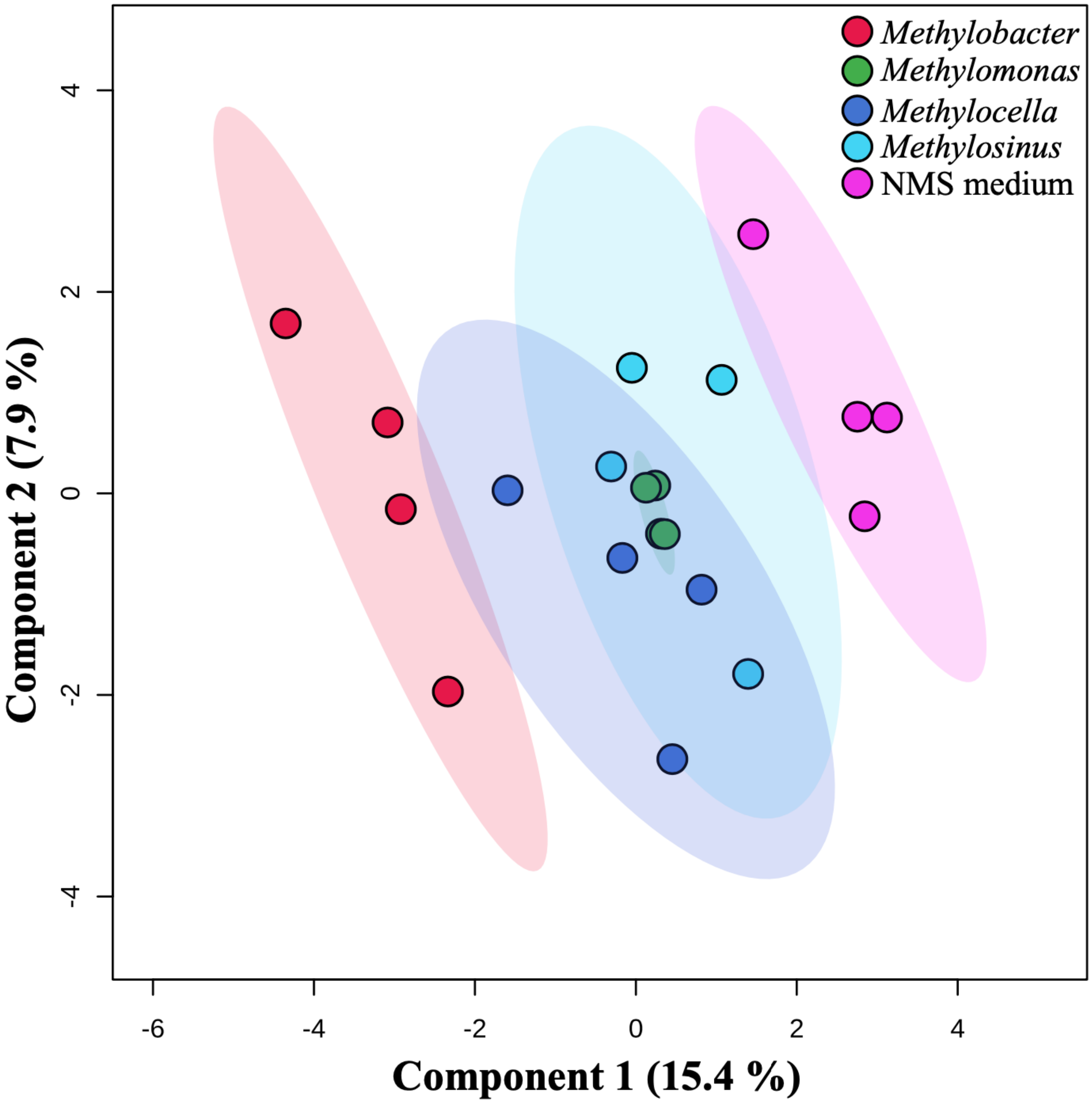

Supplementary Figure 1: PLSDA 2D-plot of GC-MS data of volatiles emitted from monocultures of MOB strains grown on NMS medium agar plates and NMS medium without inoculated bacteria.

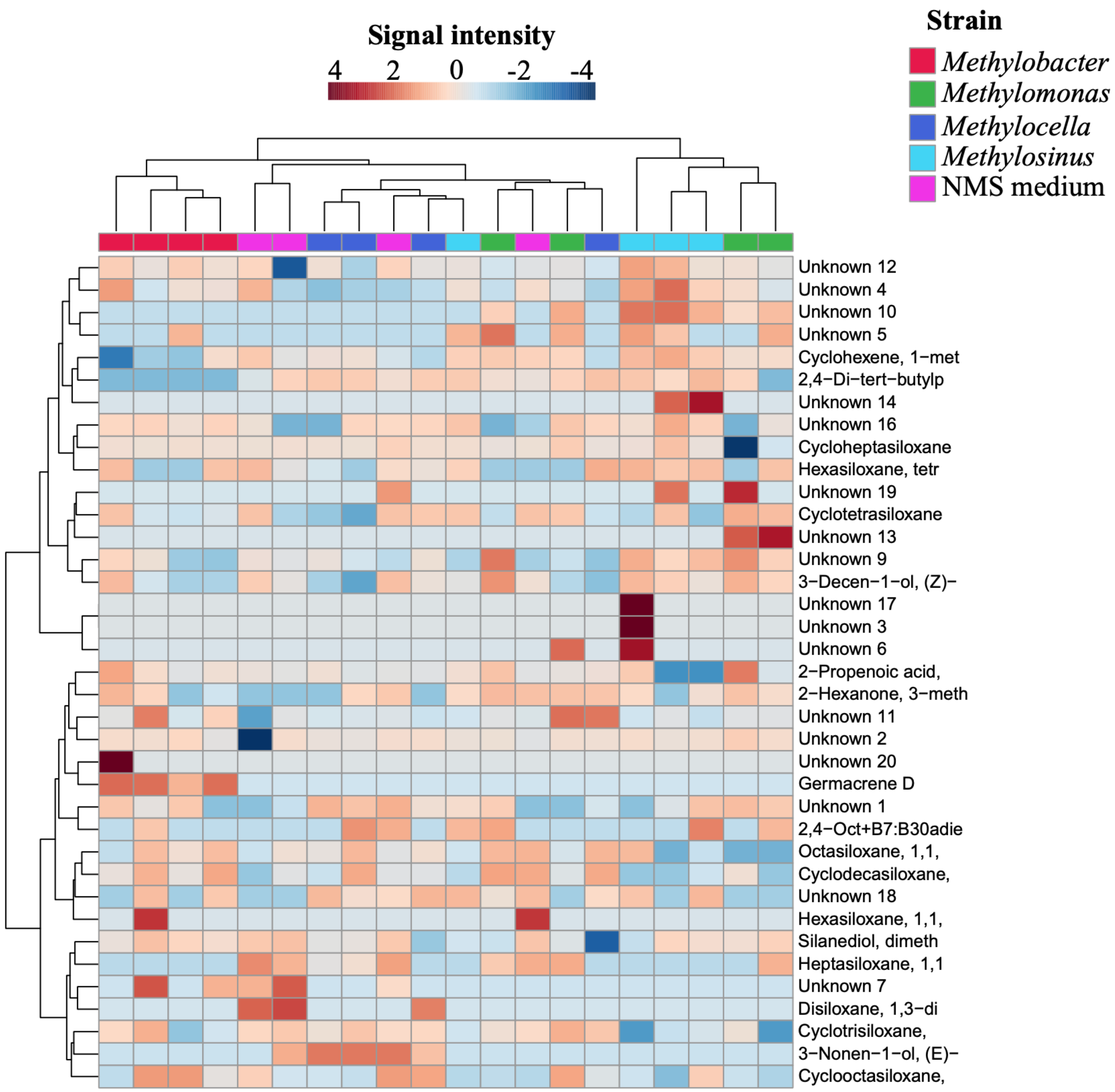

Supplementary Figure 2. A heatmap of statistically significant compounds measured in the volatolomics dataset, displaying individual sample profiles of MOB strains grown in liquid NMS medium compared to NMS medium without bacterial inoculation. The heatmap includes hierarchical clustering to group similar compounds and samples. 'Unknown' refers to compounds detected via GC-MS analysis that did not match any representative spectra in the reference databases used.

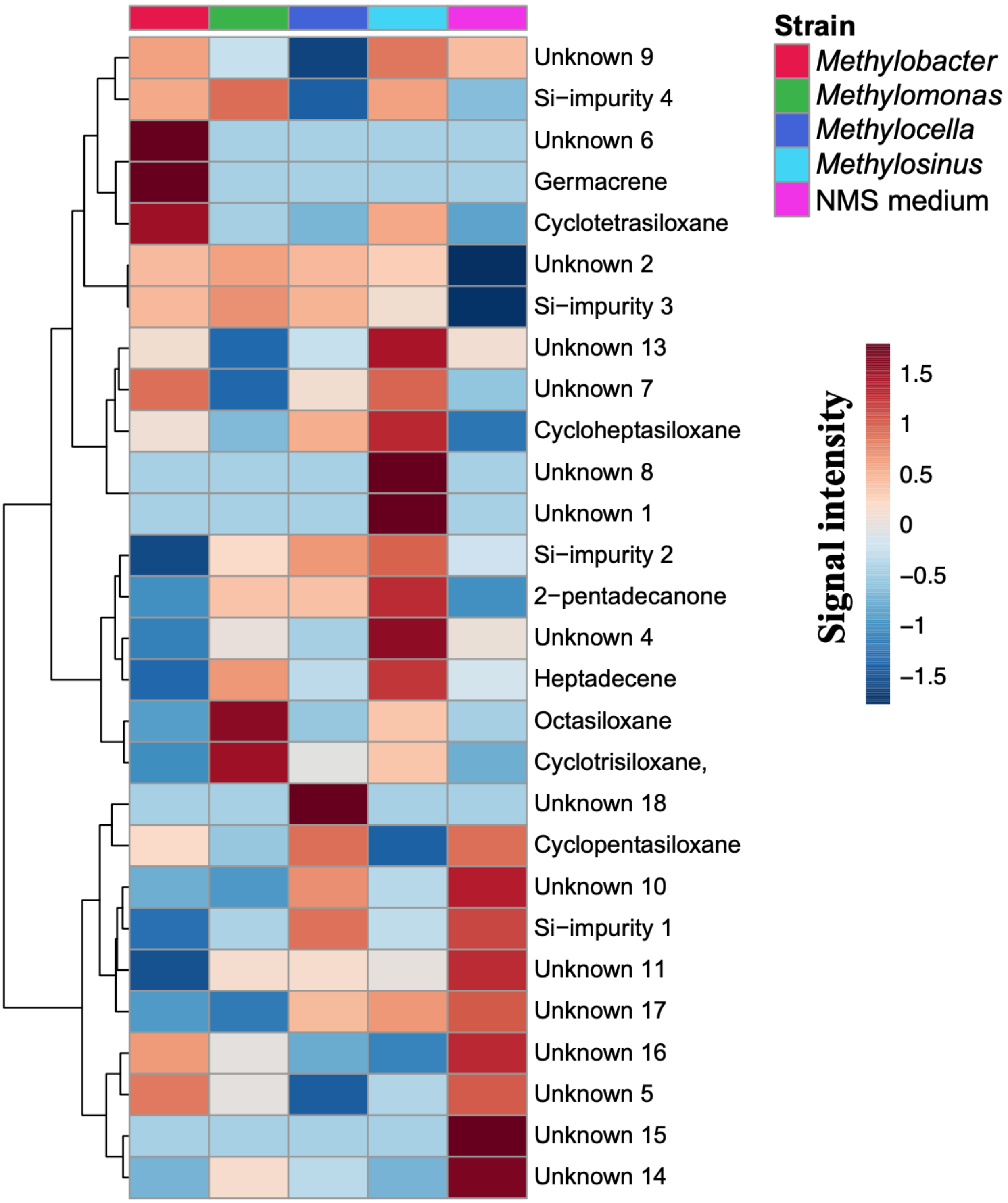

Supplementary Figure 3. A heatmap displaying average profiles of statistically significant compounds measured in the volatolomics dataset of monocultures of MOB strains grown on NMS medium agar plates, compared to NMS medium agar plates without inoculated bacteria (n=4). 'Unknown' refers to compounds identified in the GC-MS analysis but without a representative standard at the specific retention time values. 'Unknown' refers to compounds detected via GC-MS analysis that did not match any representative spectra in the reference databases used.

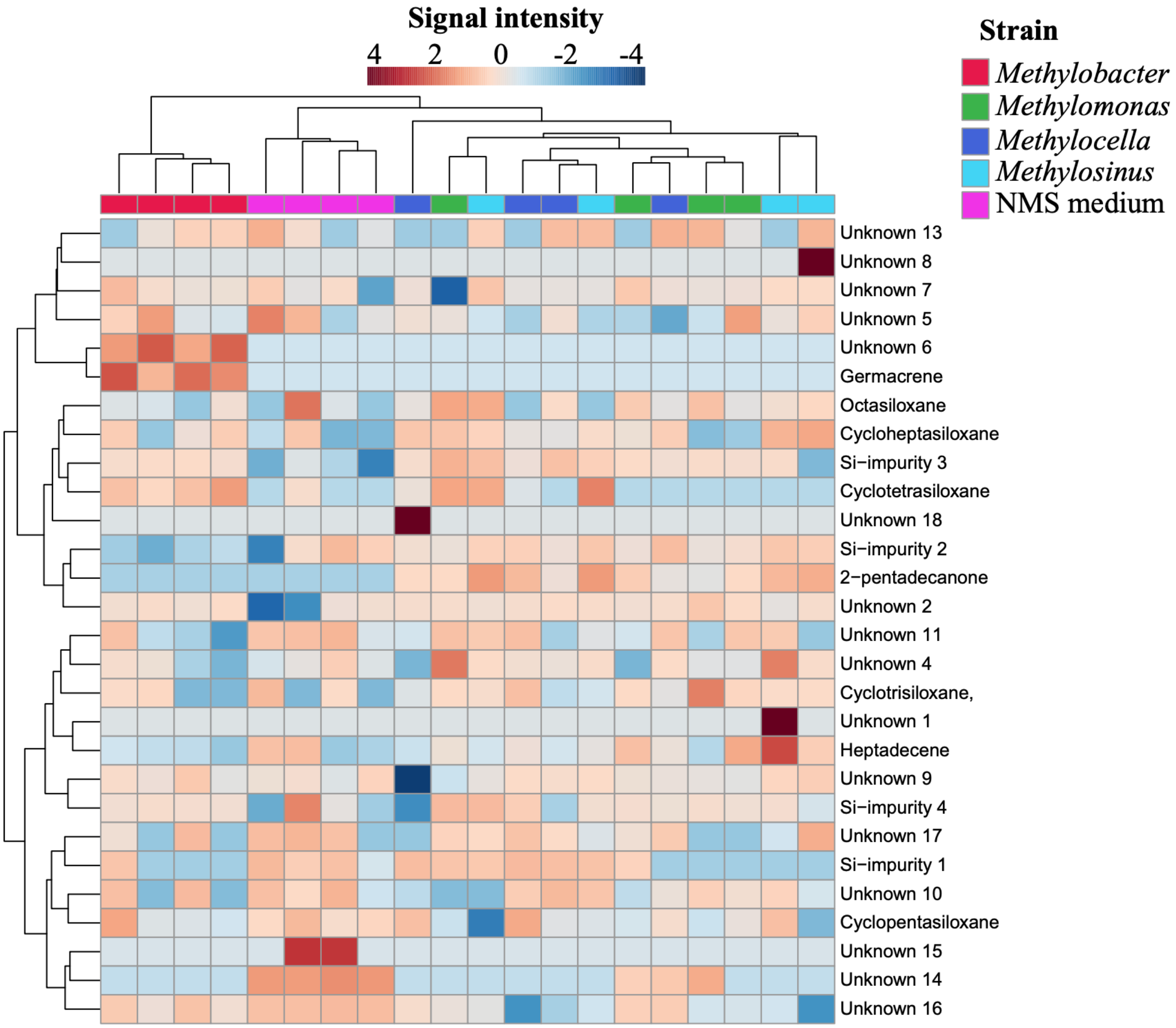

Supplementary Figure 4. A heatmap of statistically significant compounds measured in the volatolomics dataset, displaying individual sample profiles of MOB strains on NMS medium agar plates, compared to NMS medium agar plates without inoculated bacteria. The heatmap includes hierarchical clustering to group similar compounds and samples. 'Unknown' refers to compounds detected via GC-MS analysis that did not match any representative spectra in the reference databases used.

References

1. Ward N, Larsen Ø, Sakwa J, Bruseth L, Khouri H, Durkin AS, et al. Genomic insights into methanotrophy: the complete genome sequence of *Methylococcus capsulatus* (Bath). PLoS Biol. 2004;2(10).
2. Indrelid S, Kleiveland C, Holst R, Jacobsen M, Lea T. The soil bacterium *Methylococcus capsulatus* Bath interacts with human dendritic cells to modulate immune function. Front Microbiol. 2017;8:320.
3. Boden R, Cunliffe M, Scanlan J, Moussard H, Kits KD, Klotz MG, et al. Complete genome sequence of the aerobic marine methanotroph *Methylomonas methanica* MC09. 2011;193(24):7001-2.
4. Ogiso T, Ueno C, Dianou D, Van Huy T, Katayama A, Kimura M, et al. *Methylomonas koyamae* sp. nov., a type I methane-oxidizing bacterium from floodwater of a rice paddy field. Int J Syst Evol Microbiol. 2012;62(8):1832-1837.
5. Kits KD, Klotz MG, Stein LY. Methane oxidation coupled to nitrate reduction under hypoxia by the Gammaproteobacterium *Methylomonas denitrificans*, sp. nov. type strain FJG1. Environ Microbiol. 2015;17(9):3219-3232.
6. Heylen K, De Vos P, Vekeman B. Draft genome sequences of eight obligate methane oxidizers occupying distinct niches based on their nitrogen metabolism. Genome Announc. 2016;4(4).
7. de Assis Costa OY, Meima-Franke M, Bodelier PLE. Complete and draft genome sequences of aerobic methanotrophs isolated from a riparian wetland. Microbiol Resour Announc. 2021;10.
8. Hoefman S, Heylen K, De Vos P. Methylomonas lenta sp. nov., a methanotroph isolated from manure and a denitrification tank. Int J Syst Evol Microbiol. 2014;64(4):1210-1217.
9. Hur DH, Na JG, Lee EY. Highly efficient bioconversion of methane to methanol using a novel type I *Methylomonas* sp. DH-1 newly isolated from brewery waste sludge. J Chem Technol Biotechnol. 2017;92(2):311-318.
10. Hur DH, Nguyen TT, Kim D, Lee EY. Selective bio-oxidation of propane to acetone using methane-oxidizing Methylomonas sp. DH-1. J Ind Microbiol Biotechnol. 2017;44(7):1097-1105.
11. Kalyuzhnaya MG, Lamb AE, McTaggart TL, Oshkin IY, Shapiro N, Woyke T, et al. Draft genome sequences of gammaproteobacterial methanotrophs isolated from Lake Washington sediment. Genome Announc. 2015;3(2).
12. Hamilton R, Kits KD, Ramonovskaya VA, Rozova ON, Yurimoto H, Iguchi H, et al. Draft genomes of gammaproteobacterial methanotrophs isolated from terrestrial ecosystems. Genome Announc. 2015;3(3):10.1128/genomea.00515-15.
13. Flynn JD, Hirayama H, Sakai Y, Dunfield PF, Klotz MG, Knief C, et al. Draft genome sequences of gammaproteobacterial methanotrophs isolated from marine ecosystems. Genome Announc. 2016;4(1).
14. Svenning MM, Hestnes AG, Wartiainen I, Stein LY, Klotz MG, Kalyuzhnaya MG, et al. Genome sequence of the Arctic methanotroph *Methylobacter tundripaludum* SV96. J Bacteriol. 2011;193(22):6418-6419.
15. Nguyen N-L, Yu W-J, Gwak J-H, Kim S-J, Park S-J, Herbold CW, et al. Genomic insights into the acid adaptation of novel methanotrophs enriched from acidic forest soils. Front Microbiol. 2018;9:1982.
16. Khmelenina VN, Kalyuzhnaya MG, Starostina NG, Suzina NE, Trotsenko YA. Isolation and characterization of halotolerant alkaliphilic methanotrophic bacteria from Tuva soda lakes. Curr Microbiol. 1997;35(5):257-261.
17. Vuilleumier S, Khmelenina VN, Bringel F, Reshetnikov AS, Lajus A, Mangenot S, et al. Genome sequence of the haloalkaliphilic methanotrophic bacterium *Methylomicrobium alcaliphilum* 20Z. J Bacteriol. 2012;194(2):551-552.
18. Khmelenina VN, Beck DA, Munk C, Davenport K, Daligault H, Erkkila T, et al. Draft genome sequence of *Methylomicrobium buryatense* strain 5G, a haloalkaline-tolerant methanotrophic bacterium. Genome Announc. 2013;1(4).

19. Yu WJ, Lee JW, Nguyen NL, Rhee SK, Park SJ. The characteristics and comparative analysis of methanotrophs reveal genomic insights into *Methylomicrobium* sp. enriched from marine sediments. Syst Appl Microbiol. 2018;41(5):415-426.
20. Kits KD, Kalyuzhnaya MG, Klotz MG, Jetten MS, Op den Camp HJ, Vuilleumier S, et al. Genome sequence of the obligate gammaproteobacterial methanotroph *Methylomicrobium album* strain BG8. Genome Announc. 2013;1(2).
21. Medvedkova KA, Khmelenina VN, Trotsenko YA. Sucrose as a factor of thermal adaptation of the thermophilic methanotroph *Methylocaldum szegediense* O-12. Microbiology. 2007;76(4):500-502.
22. Takeuchi M, Kamagata Y, Oshima K, Hanada S, Tamaki H, Marumo K, et al. *Methylocaldum marinum* sp. nov., a thermotolerant, methane-oxidizing bacterium isolated from marine sediments, and emended description of the genus *Methylocaldum*. Int J Syst Evol Microbiol. 2014;64(Pt 9):3240-3246.
23. Wei X, Ge X, Li Y, Yu Z. Draft genome sequence of *Methylocaldum* sp. strain 14B, an obligate hydrogen sulfide-tolerant methanotrophic strain that can convert biogas to methanol. Genome Announc. 2017;5. 10.1128/genomeA.00153-17.
24. Poehlein A, Deutzmann JS, Daniel R, Simeonova DD. Draft genome sequence of the methanotrophic gammaproteobacterium *Methyloglobulus morosus* DSM 22980 strain KoM1. Genome Announc. 2013;1(6).
25. Tavormina PL, Hatzenpichler R, McGlynn S, Chadwick G, Dawson KS, Connon SA, et al. *Methyloprofundus sedimenti* gen. nov., sp. nov., an obligate methanotroph from ocean sediment belonging to the 'deep sea-1'clade of marine methanotrophs. Int J Syst Evol Microbiol. 2015;65(1):251-259.
26. Hirayama H, Fuse H, Abe M, Miyazaki M, Nakamura T, Nunoura T, et al. *Methylomarinum vadi* gen. nov., sp. nov., a methanotroph isolated from two distinct marine environments. Int J Syst Evol Microbiol. 2013;63(3):1073-1082.
27. Mateos-Rivera A, Islam T, Marshall IP, Schreiber L, Øvreås L. High-quality draft genome of the methanotroph *Methylovulum psychrotolerans* Str. HV10-M2 isolated from plant material at a high-altitude environment. Stand Genomic Sci. 2018;13(1):10.
28. Iguchi H, Yurimoto H, Sakai Y. *Methylovulum miyakonense* gen. nov., sp. nov., a type I methanotroph isolated from forest soil. Int J Syst Evol Microbiol. 2011;61(4):810-815.
29. Frindte K, Maarastawi SA, Lipski A, Hamacher J, Knief C. Characterization of the first rice paddy cluster I isolate, *Methyloterricola oryzae* gen. nov., sp. nov. and amended description of *Methylomagnum ishizawai*. Int J Syst Evol Microbiol. 2017;67(11):4507-4514.
30. Khalifa A, Lee CG, Ogiso T, Ueno C, Dianou D, Van Huy T, et al. *Methyloterrigena soli* gen. nov., sp. nov., a type I methanotroph isolated from rice field soil in Japan. Int J Syst Evol Microbiol. 2015;65(10):3527-3533.
31. Sharp CE, Smirnova AV, Kalyuzhnaya MG, Bringel F, Hirayama H, Jetten MS, et al. Draft genome sequence of the moderately halophilic methanotroph *Methylohalobius crimeensis* Strain 10Ki. Genome Announc. 2015;3(3).
32. Stein LY, Yoon S, Semrau JD, DiSpirito AA, Crombie A, Murrell JC, et al. Genome sequence of the obligate methanotroph *Methylosinus trichosporium* strain OB3b. J Bacteriol. 2010;192(24):6497-6498.
33. Belova SE, Kulichevskaya IS, Bodelier PL, Dedysh SN. Methylocystis bryophila sp. nov., a facultatively methanotrophic bacterium from acidic Sphagnum peat, and emended description of the genus *Methylocystis* (ex Whittenbury et al. 1970) Bowman et al. 1993. Int J Syst Evol Microbiol. 2013;63(3):1096-1104.
34. Han D, Dedysh SN, Liesack W. Unusual genomic traits suggest *Methylocystis bryophila* S285 to be well adapted for life in peatlands. Genome Biol Evol. 2018;10(2):623-628.
35. Vecherskaya M, Dijkema C, Saad HR, Stams AJ. Microaerobic and anaerobic metabolism of a *Methylocystis parvus* strain isolated from a denitrifying bioreactor. Environ Microbiol Rep. 2009;1(5):442-449.

36. Del Cerro C, García JM, Rojas A, Tortajada M, Ramón D, Galán B, et al. Genome sequence of the methanotrophic poly-β-hydroxybutyrate producer *Methylocystis parvus* OBBP.
37. Wartiainen I, Hestnes AG, McDonald IR, Svenning MM. *Methylocystis rosea* sp. nov., a novel methanotrophic bacterium from Arctic wetland soil, Svalbard, Norway (78N). Int J Syst Evol Microbiol. 2006;56(3):541-547.
38. Dam B, Dam S, Kube M, Reinhardt R, Liesack W. Complete genome sequence of *Methylocystis* sp. strain SC2, an aerobic methanotroph with high-affinity methane oxidation potential. J Bacteriol. 2012: 194:.https://doi.org/10.1128/jb.01446-12
39. Stein LY, Bringel F, DiSpirito AA, Han S, Jetten MS, Kalyuzhnaya MG, et al. Genome sequence of the methanotrophic alphaproteobacterium *Methylocystis* sp. strain Rockwell (ATCC 49242). J Bacteriol. 2011;193(10):2668-9.
40. Dedysh SN, Belova SE, Bodelier PL, Smirnova KV, Khmelenina VN, Chidthaisong A, et al. *Methylocystis heyeri* sp. nov., a novel type II methanotrophic bacterium possessing 'signature' fatty acids of type I methanotrophs. Int J Syst Evol Microbiol. 2007;57(3):472-479.
41. Oshkin IY, Miroshnikov KK, Dedysh SN. Draft genome sequence of *Methylocystis heyeri* H2T, a methanotroph with habitat-specific adaptations, isolated from a peatland ecosystem. Microbiol Resour Announc. 2019;8(29).
42. Lindner AS, Pacheco A, Aldrich HC, Staniec AC, Uz I, Hodson DJ. *Methylocystis hirsuta* sp. nov., a novel methanotroph isolated from a groundwater aquifer. Int J Syst Evol Microbiol. 2007;57(8):1891-1900.
43. Ghashghavi M, Belova SE, Bodelier PL, Dedysh SN, Kox MA, Speth DR, et al. *Methylotetracoccus oryzae* Strain C50C1 is a novel type Ib gammaproteobacterial methanotroph adapted to freshwater environments. mSphere. 2019;4(3).
44. In 't Zandt MH, van den Bosch TJ, Rijkers R, van Kessel MA, Jetten MS, Welte CU. Co-cultivation of the strictly anaerobic methanogen *Methanosarcina barkeri* with aerobic methanotrophs in an oxygen-limited membrane bioreactor. Appl Microbiol Biotechnol. 2018 Jul;102(13):5685-5694.
45. Tikhonova EN, Grouzdev DS, Avtukh AN, Kravchenko IK. *Methylocystis silviterrae* sp.nov., a high-affinity methanotrophic bacterium isolated from the boreal forest soil. Int J Syst Evol Microbiol. 2021 Dec;71(12). doi: 10.1099/ijsem.0.005166.
46. Dunfield PF, Khmelenina VN, Suzina NE, Trotsenko YA, Dedysh SN. *Methylocella silvestris* sp. nov., a novel methanotroph isolated from an acidic forest cambisol. Int J Syst Evol Microbiol. 2003;53(5):1231-1239.
47. Chen Y, Crombie A, Rahman MT, Dedysh SN, Liesack W, Stott MB, et al. Complete genome sequence of the aerobic facultative methanotroph *Methylocella silvestris* BL2. J Bacteriol. 2010;192(14):3840-3841.
48. Wang J, Geng K, Haque MF, Crombie A, Street LE, Wookey PA, et al. Draft genome sequence of *Methylocella silvestris* TVC, a facultative methanotroph isolated from permafrost. Genome Announc. 2018;6(8).
49. Dedysh SN, Berestovskaya YY, Vasylieva LV, Belova SE, Khmelenina VN, Suzina NE, et al. *Methylocella tundrae* sp. nov., a novel methanotrophic bacterium from acidic tundra peatlands. Int J Syst Evol Microbiol. 2004;54(1):151-156.
50. Dedysh SN, Khmelenina VN, Suzina NE, Trotsenko YA, Semrau JD, Liesack W, et al. *Methylocapsa acidiphila* gen. nov., sp. nov., a novel methane-oxidizing and dinitrogen-fixing acidophilic bacterium from Sphagnum bog. Int J Syst Evol Microbiol. 2002;52(1):251-261.
51. Tamas I, Smirnova AV, He Z, Dunfield PF. The (d) evolution of methanotrophy in the *Beijerinckiaceae*—a comparative genomics analysis. ISME J. 2014;8(2):369.
52. Dunfield PF, Belova SE, Vorob'ev AV, Cornish SL, Dedysh SN. *Methylocapsa aurea* sp. nov., a facultative methanotroph possessing a particulate methane monooxygenase, and emended description of the genus *Methylocapsa*. Int J Syst Evol Microbiol. 2010;60(12):2659-2664.

53. Dedysh SN, Didriksen A, Danilova OV, Belova SE, Liebner S, Svenning MM. *Methylocapsa palsarum* sp. nov., a methanotroph isolated from a subArctic discontinuous permafrost ecosystem. Int J Syst Evol Microbiol. 2015;65(10):3618-3624.
54. Tveit AT, Hestnes AG, Robinson SL, Schintlmeister A, Dedysh SN, Jehmlich N, et al. Widespread soil bacterium that oxidizes atmospheric methane. Proc Natl Acad Sci. 2019;116(17):8515-8524.
55. Vorob'ev AV, Baani M, Doronina NV, Brady AL, Liesack W, Dunfield PF, et al. *Methyloferula stellata* gen. nov., sp. nov., an acidophilic facultative methanotroph from Sphagnum bog. Int J Syst Evol Microbiol. 2011;61(10):2456-2463.
56. Hou S, Makarova KS, Saw JH, Senin P, Ly BV, Zhou Z, et al. Complete genome sequence of the extremely acidophilic methanotroph isolate V4, *Methylacidiphilum infernorum*, a representative of the bacterial phylum *Verrucomicrobia*. Biol Direct. 2008;3(1):26.
57. Khadem AF, Wieczorek AS, Pol A, Vuilleumier S, Harhangi HR, Dunfield PF, et al. Draft genome sequence of the volcano-inhabiting thermoacidophilic methanotroph *Methylacidiphilum fumariolicum* strain SolV.
58. Erikstad HA, Birkeland NK. Draft genome sequence of "Candidatus Methylacidiphilum kamchatkense" strain Kam1, a thermoacidophilic methanotrophic Verrucomicrobium. Genome Announc. 2015;3(2).
59. van Spanning RJM, Guan Q, Melkonian C, Gallant J, Polerecky L, Flot JF, et al. Methanotrophy by a *Mycobacterium* species that dominates a cave microbial ecosystem. Nat Microbiol. 2022;7(12):2089-2100.
